# Simultaneous Presentation of Waldenström's Macroglobulinemia and MYD88 Gene Mutation with Multiple Myeloma

**DOI:** 10.7759/cureus.3822

**Published:** 2019-01-03

**Authors:** Hassan Awada, Tariq Kewan, Fahrettin Covut, Hamed Daw, Abdo Haddad

**Affiliations:** 1 Internal Medicine, Cleveland Clinic - Fairview Hospital, Cleveland, USA; 2 Hematology and Oncology, Cleveland Clinic - Fairview Hospital, Cleveland, USA

**Keywords:** waldenström macroglobulinemia, multiple myeloma, myd88 gene mutation, b-cell neoplasms

## Abstract

Waldenström's macroglobulinemia (WM) and multiple myeloma (MM) are two distinct forms of mature hematologic B-cell malignancies. A missense somatic mutation in MYD88 gene (MYD88L265P) has been found in hematologic B-cell malignancies. The simultaneous presentation of Waldenström's macroglobulinemia and MYD88 mutation with multiple myeloma in the same patient is very rare and only a few cases have been reported in the literature.

## Introduction

Waldenström's macroglobulinemia (WM) is a rare, indolent B-cell lymphoproliferative disorder characterized by a lymphoplasmacytic infiltration in the bone marrow or lymphatic tissue and a monoclonal immunoglobulin M (IgM) protein in the serum [1-2]. It demonstrates features of both plasma cells and B lymphocytes and therefore classified as lymphoplasmacytic lymphoma. On the other hand, multiple myeloma (MM) is a multi-focal plasma-cell neoplasm characterized by serum monoclonal gammopathy in addition to other constitutional symptoms including bone lytic lesions, renal failure, anemia and/or hypercalcemia [3]. It is crucially important to distinguish between both conditions as the clinical approach and treatment to each is different.

## Case presentation

**Figure 1:**
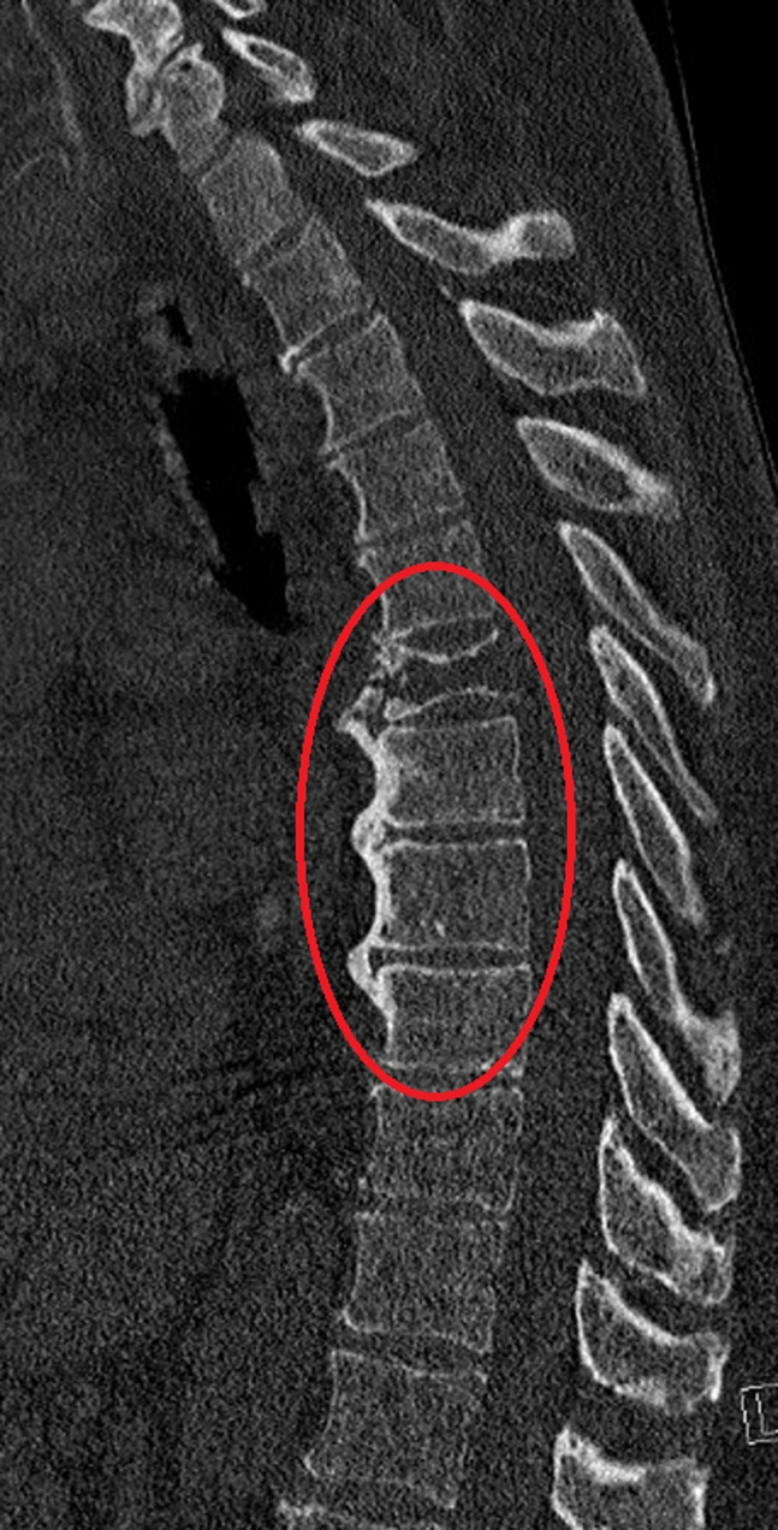
Computed tomography scan without contrast of thoracic spine A computed tomography scan without contrast of thoracic spine showing multiple myelomatous involvements of the T6 and T7 vertebra including compression deformity and ventral epidural extension at the T6 level

**Figure 2:**
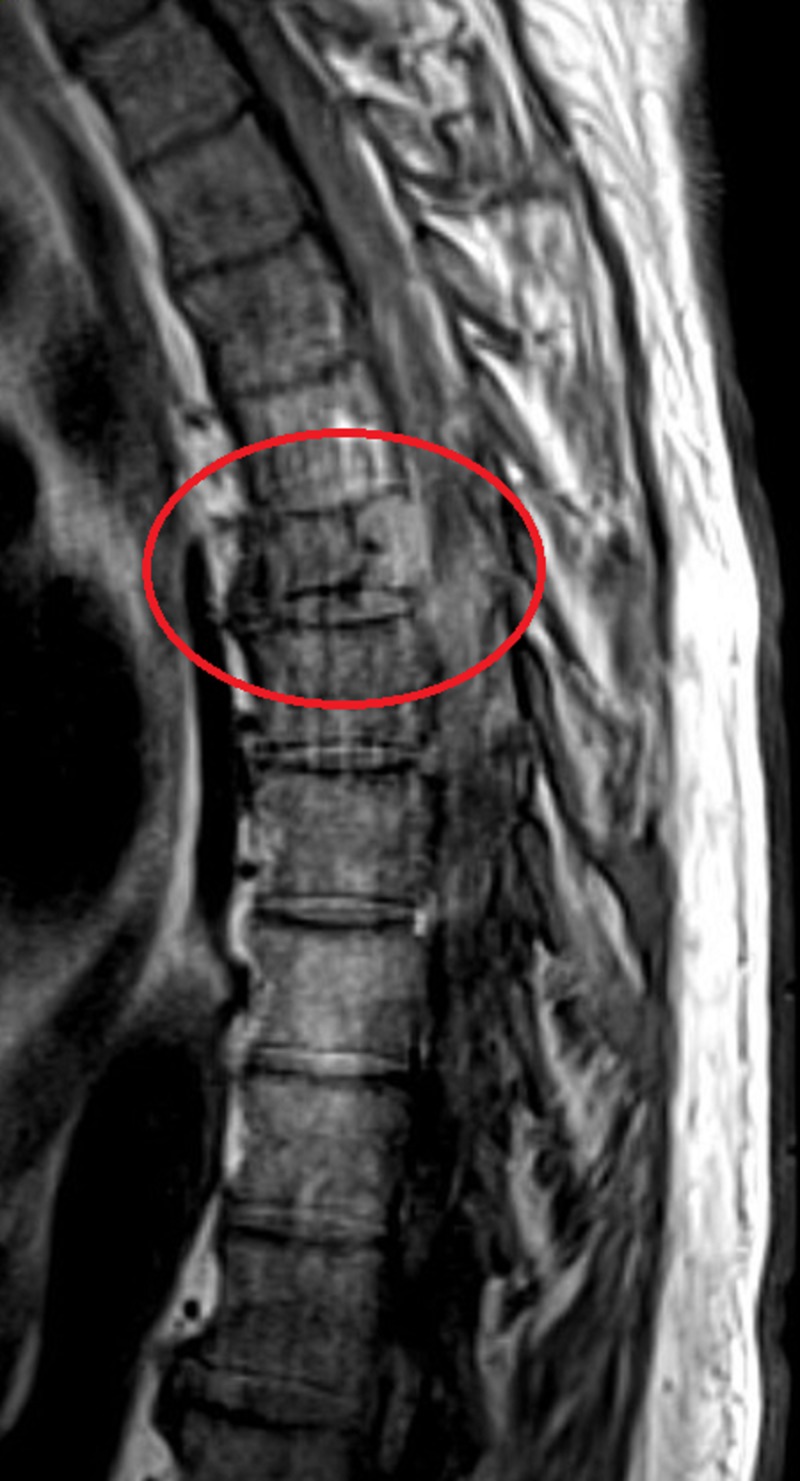
Magnetic resonance imaging of the thoracic spine with contrast A magnetic resonance imaging of the thoracic spine with contrast showing destructive osseous lesions in T6 and the transverse process on the left of T7 vertebral body

**Figure 3:**
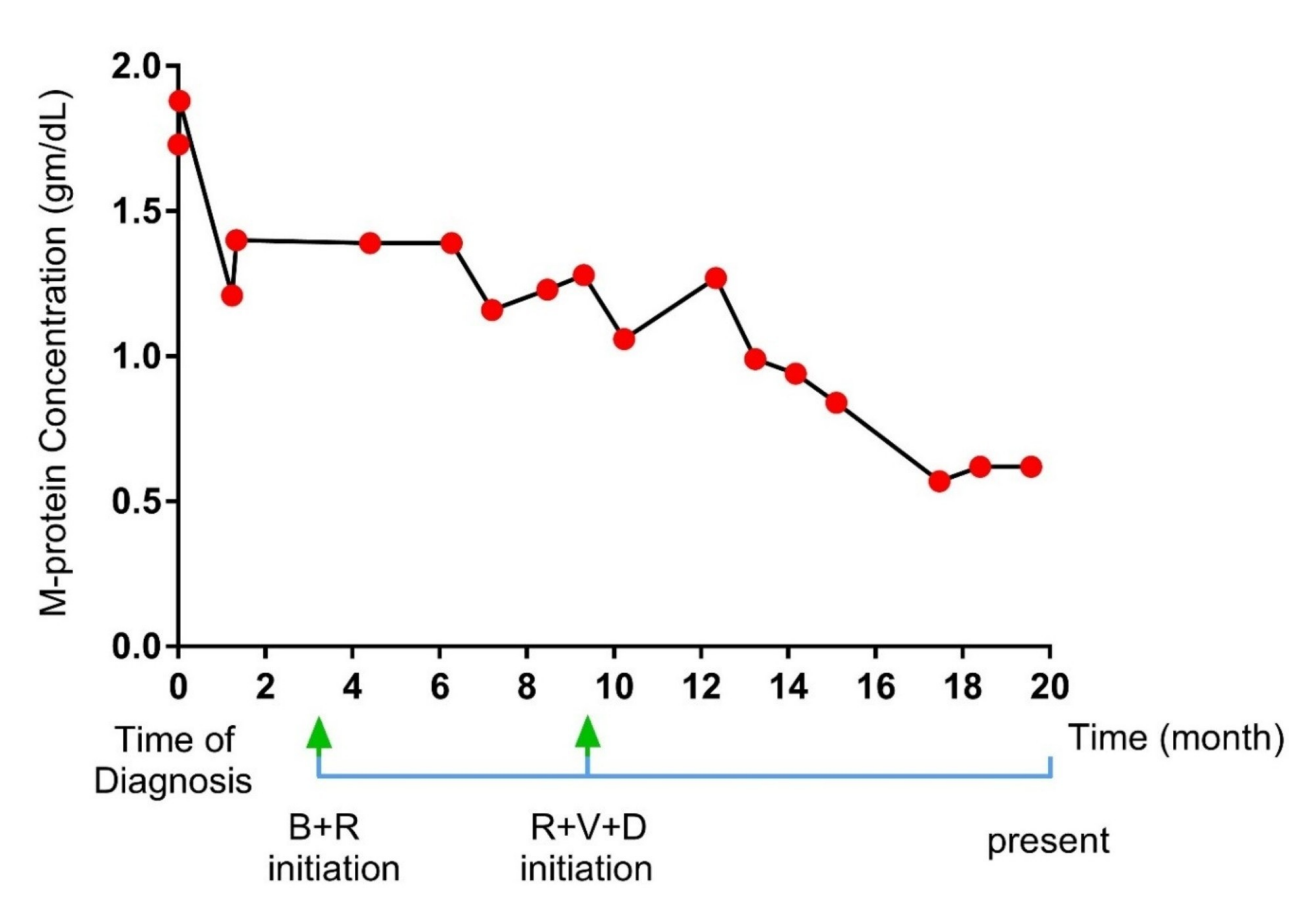
Treatment response A graph illustrating the change in M-protein concentration (g/dL) over the time (in months) of diagnosis and treatments initiation B+R: bendamustine and rituximab); R+V+D: Revlimid, Velcade and dexamethasone

A 67-year-old male with a past medical history significant for diverticulitis and hypertension presented to the emergency department for a complaint of back pain that started two days prior to admission. The patient described the pain as severe, sharp in nature and aggravates with movement. A skeletal survey reported multiple small lytic lesions. A computed tomography (CT) scan without contrast of thoracic spine showed multiple myelomatous involvements of the T6 and T7 vertebra including compression deformity and ventral epidural extension at the T6 level (Figure 1). Magnetic resonance imaging (MRI) of the thoracic and lumbar spine showed destructive osseous lesions in T6 and the transverse process on the left of the T7 vertebral body (Figure 2). CT-guided biopsy reported plasmacytoma with a negative MYD88 L265P status. M-protein concentration (1.88 mg/dL) and IgM (2,570 mg/dL) level were elevated. Serum lambda was normal (174 mg/dL), while both kappa (3,130 mg/dL) and kappa/lambda ratio (17.99) were increased. Interpretation of serum protein immunofixation electrophoresis showed biclonal gammopathy with IgM and IgG kappa light chain restriction. Flow cytometry showed no immunophenotypic evidence of involvement by a B-cell non-Hodgkin lymphoma (NHL). A subsequent bone marrow biopsy showed B-cell NHLs with plasmacytic differentiation and positive MYD88 L265P mutation. The immunostains in the core biopsy demonstrated kappa monotypic plasma cells involving approximately 5% of the marrow cellularity. Palliative radiation to T5-T9 helped improve bone lesions and pain. The patient received dexamethasone during hospitalization and was then started on a combined bendamustine and rituximab therapy. The therapy was later discontinued after a total of five cycles due to the progression of his M protein and lack of response. He was then switched to Revlimid, Velcade and dexamethasone. Consequently, his M-protein concentration started to decrease (Figure 3).

## Discussion

Interestingly, genomic data has led to the identification of a common missense somatic mutation within the MYD88 gene (MYD88L265P) found in hematologic B-cell malignancies. Subsequent investigations confirmed MYD88 mutation occurrence at high frequencies in many NHLs including the rare lymphoplasmacytic lymphoma and WM [4]. In fact, MYD88 hits were uniquely high in prevalence in WM (90% to 100% of cases) [5-7], and consequently, it facilitated as a diagnostic tool to discriminate WM from other IgM‐secreting B‐cell malignancies [8]. The MYD88 L265P is not exclusive to WM/LPL, but can also be identified, with lower prevalence, in a subset of diffuse large B‐cell lymphomas (DLBCL) of the activated B‐cell type, including splenic marginal zone lymphomas (SMZL) (4% to 13%), mucosa‐associated lymphoid tissue (MALT) lymphomas (7% to 9%) [9] and rare cases of chronic lymphocytic leukemia (CLL) [10]. However, this mutation is absent in nodal MZLs and MM [9,11].

Indeed, in our presented case, mutation detection has influenced the diagnosis of WM and the treatment initiation of bendamustine and rituximab therapy. The patient, however, did not endorse any improvement of his M-protein concentration post five cycles (Figure 3). This result has led to changing the therapy. As we started him on a new treatment with Revlimid, Velcade and dexamethasone, his M-protein concentration significantly improved (Figure 3). The latter treatment is routinely used for MM. Hence, the patient's successful response to it suggested a concurrent presentation of WM and MM that is contributing to his underlying pathology.

The simultaneous occurrence of WM and MM was present in this case. In fact, this presentation is very rare in clinical settings. Furthermore, MYD88 L265P mutation, which has been recognized as a molecular marker for WM, is not usually found in the presence of MM.

## Conclusions

Our case shows that this rare co-existence of WM and MM may occur; and molecular investigation for MYD88 mutation, on top of the clinical findings, can facilitate the recognition of such infrequent association. We, therefore, suggest that further research investigations should study this phenomenon and clarify the significance of molecular data in determining the simultaneous presentation of both diseases.
